# GreenCells: A comprehensive resource for single-cell analysis of plant lncRNAs

**DOI:** 10.1016/j.jbc.2025.110678

**Published:** 2025-09-03

**Authors:** Changxiong Wu, Jiazhi Liu, Yan Li, Wenjing Yang, Jie Wang, Changning Liu

**Affiliations:** 1 School of Life Sciences, Division of Life Sciences and Medicine, University of Science and Technology of China, Hefei, China; 2 CAS Key Laboratory of Tropical Plant Resources and Sustainable Use, Yunnan Key Laboratory of Crop Wild Relatives Omics, Xishuangbanna Tropical Botanical Garden, Chinese Academy of Sciences, Kunming, China; 3 Germplasm Bank of Wild Species & Yunnan Key Laboratory of Crop Wild Relatives Omics, Kunming Institute of Botany, Chinese Academy of Sciences, Kunming, Yunnan, China; 4 University of Chinese Academy of Sciences, Beijing, China; 5 Department of Chromosome Biology, Max Planck Institute for Plant Breeding Research, Cologne, Germany

**Keywords:** single-cell transcriptomics, lncRNAs, marker genes, database, co-expression network

## Abstract

Long non-coding RNAs (lncRNAs) play crucial roles in plant growth, development, and stress responses. With the advancement of single-cell RNA sequencing (scRNA-seq) technology, it is now possible to investigate lncRNA expression and function at single-cell resolution. Although several plant single-cell transcriptome databases have been established, they predominantly focus on protein-coding genes while largely overlooking lncRNAs. To bridge this gap, we developed GreenCells (http://greencells.liu-lab.com/), a comprehensive database specifically designed to explore plant lncRNAs at the single-cell level. By integrating scRNA-seq data from eight plant species, GreenCells has identified 2177 lncRNA marker genes and 68,869 protein-coding marker genes across diverse cell types. The database also features cell type–specific co-expression networks that reveal the regulatory potential of lncRNAs, many of which function as hub genes. For example, co-expression network analysis suggests that *lncCOBRA5* may be involved in transmembrane processes. In addition, by comparing bulk and single-cell transcriptome profiles, we identified a set of lncRNAs uniquely expressed in specific cell types that are undetectable in root bulk RNA-seq data. We also observed a strong correlation between tissue-specific and cell type–specific expression patterns of lncRNAs. Overall, GreenCells provides detailed annotations, high-quality visualizations, advanced search functions, and practical analytical tools. It offers a valuable platform for investigating the regulation and expression of plant lncRNAs at single-cell resolution, thereby contributing a new resource to plant functional genomics research.

Long non-coding RNAs (lncRNAs) are RNA molecules longer than 200 nucleotides that do not encode proteins (1). Initially regarded as transcriptional noise with no functional significance (2), lncRNAs have now been recognized for their critical roles in regulating gene transcription, orchestrating plant development and cell differentiation, and mediating responses to environmental stresses such as drought, salinity, and low temperatures (3, 4, 5, 6). For example, in Arabidopsis, the lncRNA *COLDAIR* regulates flowering time by interacting with the Polycomb Repressive Complex 2 (PRC2) to suppress *FLC* expression (7). In addition, several functionally characterized lncRNAs have been identified, such as *IPS1*, which regulates phosphate homeostasis (8); *ASCO-lncRNA*, which modulates lateral root development (9); and *APOLO*, which influences plant development through auxin signaling pathways (10). These findings highlight the significant biological functions of lncRNAs in plant growth, development, and environmental adaptation, making them a focal point in functional genomics research.

Despite the rapid development of high-throughput sequencing technologies and the establishment of plant-specific lncRNA databases like PLncDB (11) and GreenNC (12), our understanding of plant lncRNAs functions remain limited. This limitation arises from several challenges, including the low sequence conservation of lncRNAs, which hampers cross-species functional inferences (13), and their typically low expression levels (14), making detection and functional characterization difficult. Computational approaches, including co-expression network analysis, have been increasingly employed to predict lncRNAs functions. For example, six novel lncRNAs identified through co-expression analysis may serve as biomarkers for cutaneous squamous cell carcinoma by regulating *ACY3*, *NR1D1*, and *MZB1*, potentially influencing apoptosis and autophagy. Similarly, *MSTRG.8888.1* was identified as a hub gene in co-expression module and affected the expressions of some salt tolerance–related functional transcripts (15). In cucumber, a lncRNA–mRNA co-expression network also revealed lncRNAs involved in stress response regulation (16).

While bulk RNA sequencing (RNA-seq) has been a powerful tool for transcriptomic analysis (17), its averaging of transcripts across diverse cell populations can obscure lncRNAs that are restricted to specific cell types or cellular states (18). The advent of single-cell RNA sequencing has revolutionized transcriptomics by providing high-resolution data that enables the study of cellular diversity and dynamic developmental processes (19, 20). Given the cell-specific nature of lncRNA expression, scRNA-seq presents a powerful method for investigating the regulation of lncRNAs at single-cell resolution (18). For instance, a study on COVID-19 patients identified the myeloid-specific lincRNA *PIRAT* in CD14^+^ monocytes (21). Similarly, scRNA-seq analysis revealed that the lncRNA *Maenli* is co-expressed with *En1* in embryonic cells and activates its expression through cis-regulation, contributing to limb development in both mice and humans (22). Furthermore, See *et al.* used single-cell data to identify 359 cardiac nuclear lincRNAs, 30% of which were previously unannotated.

To promote the study of plant lncRNAs at the single-cell level, developing a dedicated plant single-cell database focused on lncRNAs is of great significance. Although several plant-specific single-cell databases have been established, such as PlantscRNAdb (23), scPlantDB (24), and PsctH (25), these resources primarily emphasize protein-coding genes. As a result, a specialized database centered on plant lncRNAs at single-cell resolution has been urgently needed. To address this gap, we have developed a comprehensive platform designed to explore the expression patterns and regulatory characteristics of plant lncRNAs at single-cell resolution. This platform systematically compiles scRNA-seq data from eight plant species, including *Arabidopsis thaliana*, *Oryza sativa*, *Zea may* and *Solanum lycopersicum*, and others, covering 14 different tissue types. With consistent quality control and data processing, we have identified numerous cell-type-specific lncRNAs and protein-coding genes, all of which are accessible through our online platform. The database also provides cell-type-specific co-expression networks that include both lncRNAs and protein-coding genes and enabling deeper investigations of lncRNAs. Further analyses revealed that many lncRNAs play key roles in cellular regulatory networks, with some acting as hub genes, suggesting their potential involvement in cellular function regulation and signal transduction. Compared to PscLncRNA (26), a recently published plant single cell transcriptome database, our platform is more comprehensive. It offers a broader range of plant species, incorporates both merged and individual sample analyses, and enables more in-depth exploration of lncRNA expression and network characteristics across diverse samples. Additionally, we have integrated useful tools such as single-cell data analysis modules, BLAST, and network visualization tools to facilitate in-depth lncRNA mining. We are confident that our database will serve as an important resource for advancing our understanding of plant lncRNAs and their diverse biological functions at single-cell resolution.

## Results

### GreenCells platform overview and main functions

**Figure 1:**
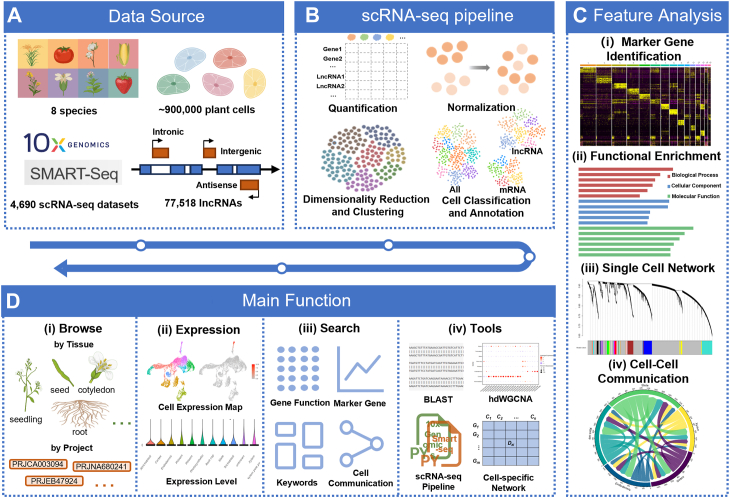
**The workflow of GreenCells.***A*, basic data statistics included in the database, covering eight species, approximately 900,000 cells, and over 77,000 lncRNAs. *B*, general workflow for upstream analysis of single-cell data. *C*, key features of the database, including marker gene identification, functional enrichment analysis, cell type-specific co-expression networks, and cell–cell communication analysis. *D*, main modules of the GreenCells database: Browse, Expression, Search, and Tools.

GreenCells is an integrative platform designed for studying lncRNAs using plant single-cell transcriptomic data. Our work can be broadly divided into four major components: (i) data collection, (ii) transcriptome quantification and clustering analysis, (iii) advanced analyses, and (iv) database construction (Fig. 1).

**Figure 2:**
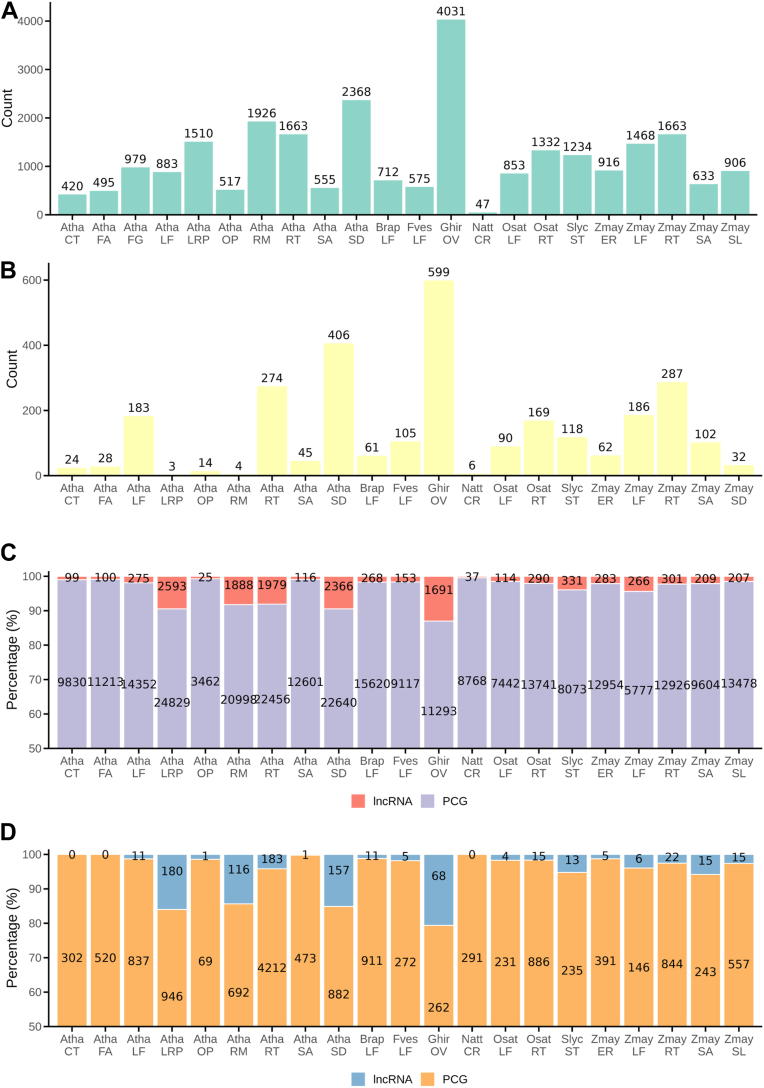
**LncRNAs related statics.***A*, expression counts of lncRNAs across different tissues. *B*, predicted lncRNA marker gene counts. *C*, number of genes in co-expression network modules for each tissue, including lncRNAs and protein-coding genes (PCGs). *D*, number of hub genes in co-expression network modules for each tissue, including lncRNAs and protein-coding genes (PCGs).

The first step involved comprehensive data collection. The data were obtained from previously published studies. Specifically, we curated 39 high-quality plant scRNA-seq studies by systematically searching Google and NCBI using keywords such as “single cell transcriptomics” and “scRNA-seq”. These studies cover eight plant species and 14 tissue types, comprising approximately 4690 samples and 900,000 cells, with most data generated using the 10× Genomics platform (Fig. 1*A*). A key focus of this study was the incorporation of lncRNAs. We collected approximately 125,428 lncRNAs from PLncDB, NCBI, and relevant publications, removing those overlapping with protein-coding genes to ensure accurate quantification, and remained about 77,518 lncRNAs. These lncRNAs were categorized as intronic (2.88%), intergenic (69.88%), or antisense (27.24%), the lengths of lncRNA genes vary across species (Fig. S1a); however, most lncRNA genes contain only a single transcript (Fig. S1*B*). These transcripts exhibit diverse exon counts, with the majority containing between one and five exons (Fig. S1*C*). Subsequently, all lncRNAs were integrated into species-specific reference genomes and incorporated into the corresponding annotation files (Fig. 1*A*). Comprehensive lncRNA lists can be accessed through the “Download” section of the database. Building upon the curated data, we performed transcriptome quantification and downstream analyses, including normalization, dimensionality reduction, clustering, and cell type annotation, based on expression matrices that incorporated lncRNAs (Fig. 1*B*). Our analysis revealed widespread expression of lncRNAs across diverse plant tissues (Fig. 2*A*), and these lncRNAs exhibit varying levels of expression across different species (Fig. S1*D*). Notably, substantial variation in lncRNA expression was observed across species and among different tissues. For example, *Gossypium hirsutum* expressed the highest number of lncRNAs (4,031), whereas *Nicotiana* attenuata expressed only 47 (Fig. 2*A*). These differences may, in addition to species-specific expression variation, reflect differences in the number of lncRNAs included in the annotation files of each species. Even within a single species, such as *A. thaliana*, distinct tissues exhibited varying levels of lncRNA expression—for instance, 420 lncRNAs were detected in cotyledon, compared to 2368 in seed (Fig. 2*B*). Protein-coding genes also showed variability in expression patterns across tissues and species (Fig. S2*A*).

To further characterize the single-cell transcriptomic profiles and explore potential functional roles of lncRNAs, we performed comprehensive downstream analyses such as marker gene identification, functional enrichment, co-expression network construction, and inference of cell–cell communication (Fig. 1*C*). We performed *de novo* marker gene prediction and identified 2177 marker lncRNAs (Figs. 2*B*) and 68,869 marker mRNAs (Fig. S2*B*). *G. hirsutum* exhibited the highest number of lncRNA markers (599), whereas *N.* Nicotiana attenuata had the fewest (6) (Fig. 2*B*). In *A. thaliana*, seeds showed the highest number of lncRNA markers (406), followed by roots (274) and leaves (183) (Fig. 2*B*). Marker prediction was conducted on both individual and merged datasets, and final counts representing non-redundant results. While the number of markers varied across species and tissues, no consistent pattern was observed. To further characterize these marker lncRNAs, we assessed their cell-type specificity through downstream analyses. In addition to conventional cell-type annotation based on known marker genes, we performed Gene Ontology (GO) enrichment analysis for each cell cluster to gain functional insights. Building on the annotated cell types, we then applied hdWGCNA (27) to construct cell-type-specific co-expression networks, identifying 3817 modules, many of which were enriched in lncRNAs (Fig. 2*C*). Notably, several lncRNAs functioned as hub genes within these modules (Fig. 2*D*), indicating their potential regulatory significance. Our analysis further revealed that hub lncRNAs exhibit significantly higher expression levels than randomly selected lncRNAs (Fig. S3*A*), a trend also observed for protein-coding hub genes (Fig. S3*B*). This finding is based on transcriptomic data from Arabidopsis roots (data sources provided in Table S1). Notably, unlike protein-coding hubs, lncRNA hubs display a bimodal expression pattern. This intriguing observation may reflect the functional versatility of lncRNA hubs, aligning with the well-established mechanistic diversity by which lncRNAs exert their biological functions (28). Extending beyond gene co-expression, we used PlantPhoneDB (29) to infer cell–cell communication across cell types, resulting in a comprehensive analysis of ligand–receptor interactions across a wide range of plant scRNA-seq datasets. This analysis revealed 119,640 putative ligand–receptor interactions between cell types (Fig. S2*C*), providing a valuable resource for understanding intercellular signaling dynamics in plants.

**Figure 3:**
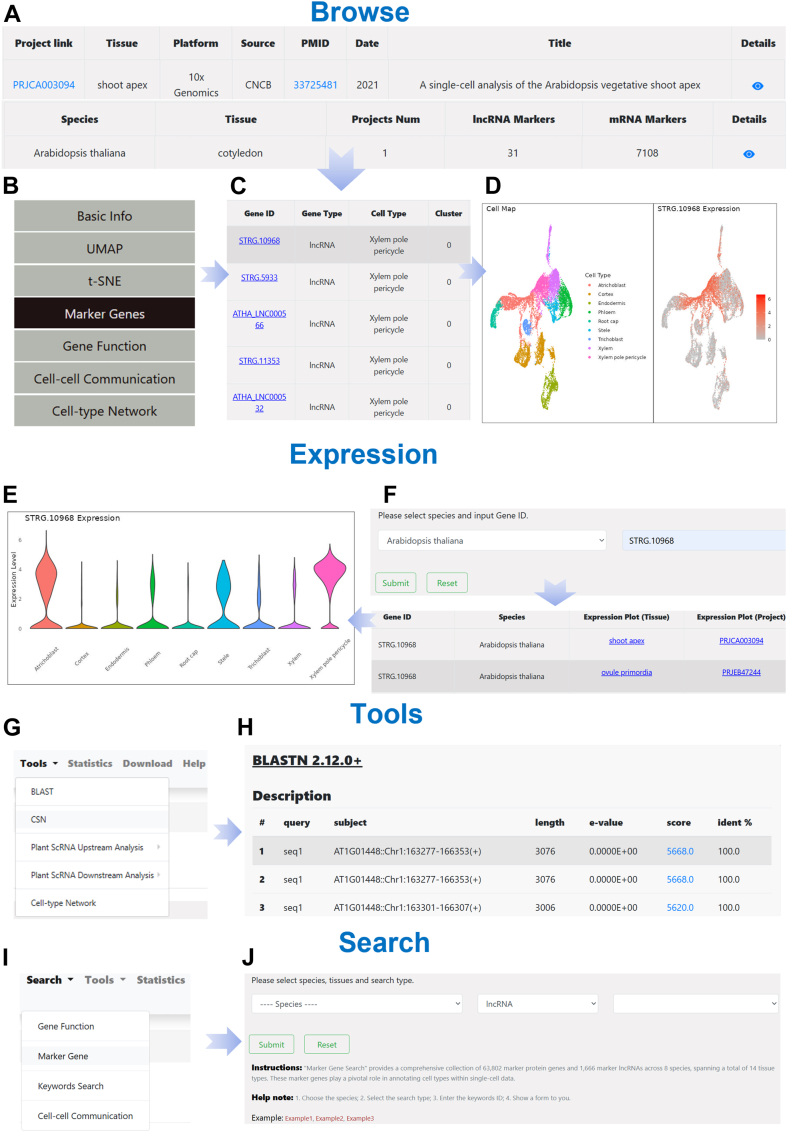
**Main modules displayed on the GreenCells website.***A*, detailed visualization of analysis results, supporting both single-dataset insights and cross-dataset integration. *B*, the content selection *panel* in the Browse module includes a sidebar that enables users to explore various data categories, such as sample metadata, clustering visualizations (t-SNE and UMAP), predicted marker genes, enrichment results, cell-cell communication analysis, and cell type–specific co-expression networks. *C*, A comprehensive list of marker genes, including both lncRNAs and protein-coding genes, along with their associated cell types, expression proportions across different cell populations, and statistical significance (*e.g.*, *p*-values). *D*, UMAP plots visualizing the expression patterns of selected marker genes. Violin plots, as shown in (*E*), are also available for expression comparison. *E* and *F*, the Expression module allows users to select a species and search for specific genes to examine their expression patterns across different datasets and tissues. Gene expression can be visualized using violin plots or UMAP plots, as shown in (*D*). *G* and *H*, the Tools module provides five functional tools (*G*), including a sequence alignment tool, a single-cell network construction tool, upstream/downstream gene analysis tools, and a network module extraction tool. Users can access each tool by clicking the corresponding button—for example, clicking the BLAST tool initiates a sequence alignment, as shown in (*H*), where the input sequence is aligned to the *AT1G01448* gene. *I* and *J*, the Search module provides four search options: functional enrichment search, marker gene search, keyword search, and cell–cell communication result search. Users can choose different options based on their needs. For example, as shown in (*J*), the marker gene search allows users to filter by species, tissue, and gene type to retrieve marker genes of interest.

Finally, all analytical results were integrated into a user-friendly and accessible web interface. The resulting database is organized into four main functional modules: Browse, Expression, Search, and Tools (Fig. 1*D*). The “Browse” module presents analysis results from both single-sample and integrated multi-sample approaches (Fig. 3*A*), featuring clustering visualizations, marker genes (lncRNAs and protein-coding), GO enrichment, cell-cell communication, and cell-type–specific co-expression networks (Fig. 3, *B* and *C*). The “Expression” module allows users to explore gene expression across species, tissues, or datasets, visualized *via* UMAP and violin plots (Fig. 3, *D*–*F*). Given the substantial computational resources required, the current “Expression” module is limited to exploration of pre-computed results rather than supporting online single-cell transcriptome analysis. The “Tools” module offers key utilities including BLAST, the CSN tool, a single-cell data analysis script generator, and a network module extractor (Fig. 3, *G* and *H*). The BLAST tool aligns user-submitted sequences to database genes, enabling exploration of matched gene expression and networks. The CSN tool constructs single-cell gene co-expression networks (30). Users can also generate customized R scripts for single-cell analysis or extract network modules based on parameters such as correlation thresholds and gene types. The “Search” module supports multiple query types, such as enrichment results, marker genes, keywords, and cell–cell communication data (Fig. 3, *I* and *J*)—making it easier for users to navigate and utilize the database effectively. Together, these modules make GreenCells a comprehensive and user-friendly resource for investigating plant lncRNA expression and co-expression networks at single-cell resolution.

### Specific expression of marker genes

Marker genes play a crucial role in identifying specific cell types, particularly in single-cell transcriptomic analyses (31), where reliable marker genes are essential for accurate cell-type annotation. Through the collection and curation of published literature and database, we compiled approximately 1055 well-characterized marker genes, spanning 15 tissues, seven species, and about 80 distinct cell types, sourced from 37 publications, which are all accessible in the “Download” module in the GreenCells database. In addition to curating well-characterized marker genes, we also predicted novel markers, encompassing both lncRNAs and protein-coding genes. To further assess their cell-type specificity at the single-cell level, we analyzed their expression patterns using the Arabidopsis root dataset as a representative example.

**Figure 4:**
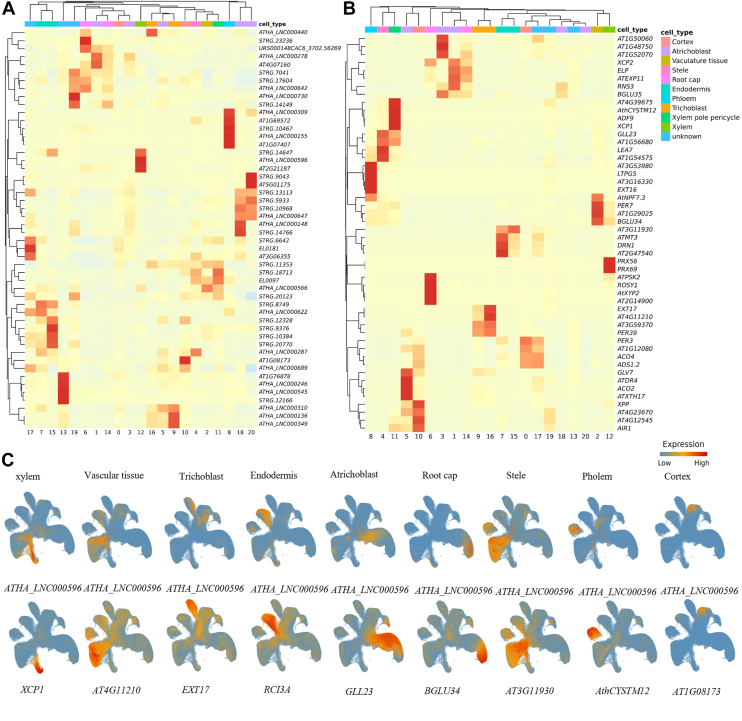
Expression patterns of predicted root marker genes (*A*) Heatmap showing the expression patterns of predicted lncRNA marker genes across cell clusters, displaying the top 50 genes with normalized expression values. *B*, Heatmap showing the expression patterns of predicted protein-coding marker genes across cell clusters, displaying the top 50 genes with normalized expression values. *C*, comparison of the expression localization of predicted lncRNA and protein-coding marker genes. lncRNAs are shown at the top, followed by protein-coding genes, with cell type labels indicated above.

First, we integrated multiple scRNA-seq datasets of Arabidopsis root (data sources listed in Table S1) and performed clustering and annotation. As a result, 21 cell clusters were identified and subsequently annotated into major cell types such as atrichoblast, xylem, phloem, cortex, and endodermis, based on the expression patterns of known marker genes (Fig. S4). Next, we conducted marker gene prediction and identified a total of 121 lncRNA markers and 11,496 protein-coding gene markers. We observed that the lncRNA markers exhibited distinct and robust cell-type-specific expression patterns. For instance, *STRG.6642*, *EL0181*, and *AT3G06355* showed clear cluster-specific expression in cluster 17 (Fig. 4*A*). Several lncRNAs also exhibited notable specificity in cluster 8 (Fig. 4*A*). The remaining lncRNAs also displayed distinct cell-cluster-specific expression patterns, highlighting their potential as novel markers for cell-type identification (Fig. 4*A*). Consistently, protein-coding genes also demonstrated pronounced cell-type specificity (Fig. 4*B*). Interestingly, some of the predicted coding marker genes overlapped with well-characterized markers, such as *XCP1*, *XCP2* (32), *XTH17* (33), and *XPP* (34).

Furthermore, we observed that some lncRNA markers exhibited expression patterns highly consistent with those of protein-coding markers (Fig. 4*C*). For instance, both the lncRNA *ATHA_LNC000596* and the protein-coding gene *XCP1* showed strong and specific expression in xylem cells (Fig. 4*C*). *XCP1* is a well-known marker in xylem, and recent studies have demonstrated its conservation across various plant species (35). Similarly, the known protein-coding marker *RCI3A* (32) also exhibited co-expression with *ATHA_LNC000596* in endodermis cells. To further assess the reliability of the predicted lncRNA markers, we focused on cell types for which a relatively large number of lncRNA markers were identified. For each of these cell types, we calculated the Spearman correlation between the predicted lncRNA markers and the known marker genes (see Table S2) based on their expression profiles across all single cells. The results showed that the predicted lncRNA markers exhibited significantly stronger expression correlations with known markers compared to the randomly selected non-marker lncRNAs (Fig. S5, *A*–*F*). For example, in xylem cells, predicted lncRNA markers showed peak correlation (∼0.5) with known markers, whereas non-marker lncRNAs peaked near 0.01 (Fig. S5*A*). These findings suggest that the predicted lncRNA markers may share similar expression patterns with known marker genes, supporting their potential relevance in defining cell identity.

### Comparison of cell-specific expression patterns

**Figure 5:**
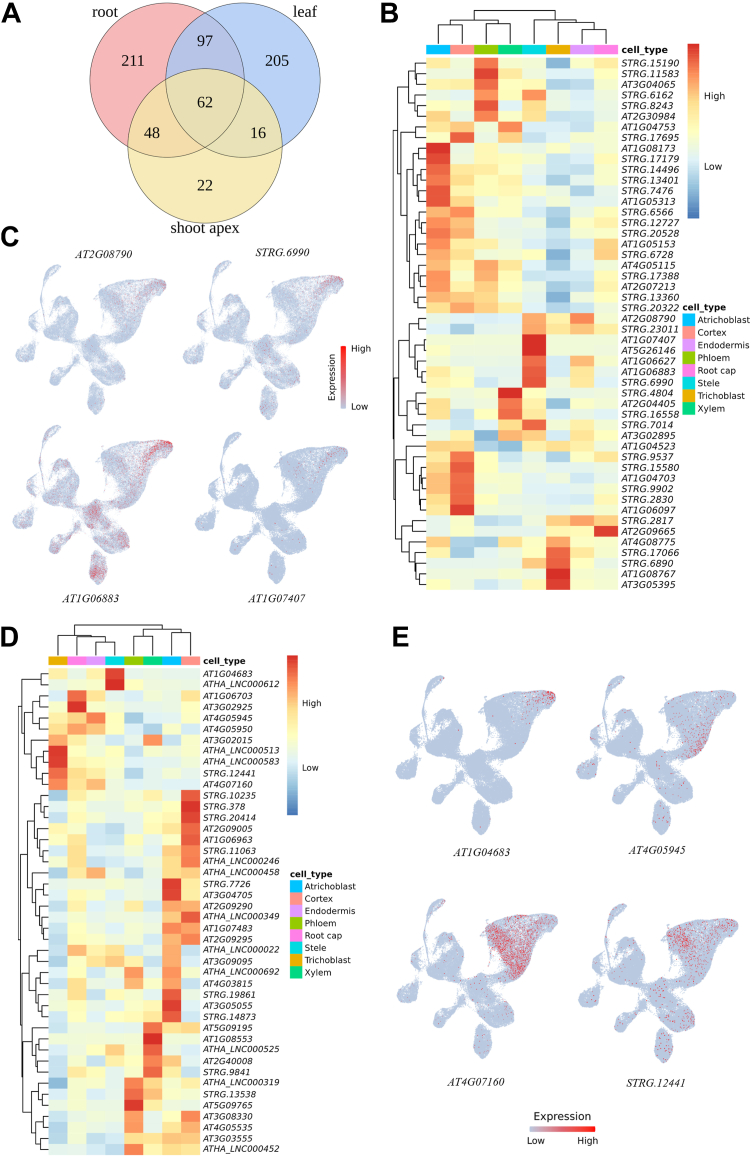
**Comparison of lncRNA expression patterns between single-cell RNA-seq and bulk RNA-seq data.***A*, some lncRNAs that were undetected in six bulk transcriptome datasets were found to be expressed in single-cell data from leaf, root, and shoot apex tissues. *B*, Heatmap showing the expression patterns of lncRNAs that are absent in bulk root tip data of *Arabidopsis thaliana* but detected in scRNA-seq data. The figure shows the top 50 lncRNAs based on average expression level after being normalized. *C*, UMAP plot showing the expression of the top four lncRNAs specifically expressed in the stele, as identified in (*B*). *D*, Heatmap showing the expression patterns of lncRNAs specifically detected in bulk root tip data across cell types in the scRNA-seq data of *Arabidopsis thaliana*. The figure shows the top 50 lncRNAs based on average expression level after being normalized. *E*, UMAP plot showing the expression of the top four lncRNAs specifically enriched in the root cap, as identified in (*B*).

Bulk RNA sequencing (RNA-seq) provides gene expression profiles by averaging signals across all cells within a tissue, making it challenging to detect genes expressed in rare cell populations (36). Given that most lncRNAs exhibit strong cell-type specificity (37), RNA-seq alone may be insufficient to study their functions. In contrast, scRNA-seq preserves gene expression heterogeneity and may offer deeper insights into lncRNAs as it preserves gene heterogeneity. Based on this, we performed a comprehensive comparative analysis to better understand lncRNAs in various contexts. We first analyzed RNA-seq data from multiple *Arabidopsis* tissues, including the root tip, stem, leaf, silique, flower, and bud (data sources listed in Table S3). The results showed that approximately 2365 lncRNAs were not detectable in the bulk tissue samples. However, scRNA-seq analysis revealed that many of these "undetected" lncRNAs were actually expressed (counts >1, in at least three cells) in root (38), leaf (39), and shoot apex (40) (Fig. 5*A*), with the highest number (419 lncRNAs) detected in the root tip.

To gain deeper insights into this phenomenon, we conducted a focused analysis on the root tip. Among approximately 3000 lncRNAs that were undetectable by bulk RNA-seq, 714 were found to be expressed at the single-cell level, underscoring the increased sensitivity of scRNA-seq in capturing low-abundance or highly localized transcripts. Interestingly, these lncRNAs exhibited pronounced cell-type-specific expression patterns, with the highest number specifically enriched in atrichoblast cells and the fewest in root cap cells (Fig. 5*B*). A similar distribution pattern was observed for protein-coding genes (Fig. S6), reinforcing the notion that scRNA-seq can identify lncRNAs and protein-coding genes with cell-type-specific expression profiles. To further characterize the expression features of these lncRNAs detectable only by scRNA-seq, we selected the top four lncRNAs showing stele-specific expression. Analysis of their expression profiles revealed highly restricted and localized patterns (Fig. 5*C*). For example, *AT1G06883*, the most abundantly expressed among them, was detected in only 6.98% of all single cells, while *AT1G07407* was detected in merely 0.78% of cells. These findings collectively suggest that many lncRNAs, although present at appreciable levels within specific cell populations, are missed in bulk RNA-seq due to signal dilution across heterogeneous tissues.

Building on these observations, we aimed to assess whether tissue-specific lncRNAs also exhibit cell-type specificity at the single-cell level. To this end, we identified 109 root tip-enriched lncRNAs and 227 protein-coding genes based on RNA-seq data from six Arabidopsis tissues (Table S2). ScRNA-seq analysis revealed that most of these lncRNAs displayed pronounced cell-type-specific expression. For example, the top 50 lncRNAs were predominantly specifically expressed in cortex and atrichoblast cells, with fewer lncRNAs exhibiting specific expression in other cell types (Fig. 5*D*). Moreover, UMAP plots of lncRNAs such as *AT1G04683*, *AT4G05945*, *AT4G07160*, and *STRG.12441* confirmed their localized expression within distinct root cell types (Fig. 5*E*). These findings suggest that the tissue-specific expression of lncRNAs is primarily driven by their cell-type specificity, highlighting the value of single-cell resolution in capturing these intricate expression patterns that would otherwise be diluted in bulk RNA-seq analyses (41). This distribution closely mirrored the expression patterns of protein-coding genes (Fig. S7), indicating that both the tissue-specific lncRNAs and protein-coding genes follow similar cell-type-specific expression patterns.

In summary, scRNA-seq enables the detection of lncRNAs that are expressed in only a subset of cells, overcoming the averaging effect of bulk RNA-seq. Additionally, scRNA-seq provides a more refined resolution of tissue-specific lncRNAs, revealing their further specialization at the cellular level. These findings highlight the unique advantage of scRNA-seq in precisely characterizing lncRNA expression patterns and functions at single-cell resolution.

### Predicting lncRNAs functions based on single-cell co-expression networks

**Figure 6:**
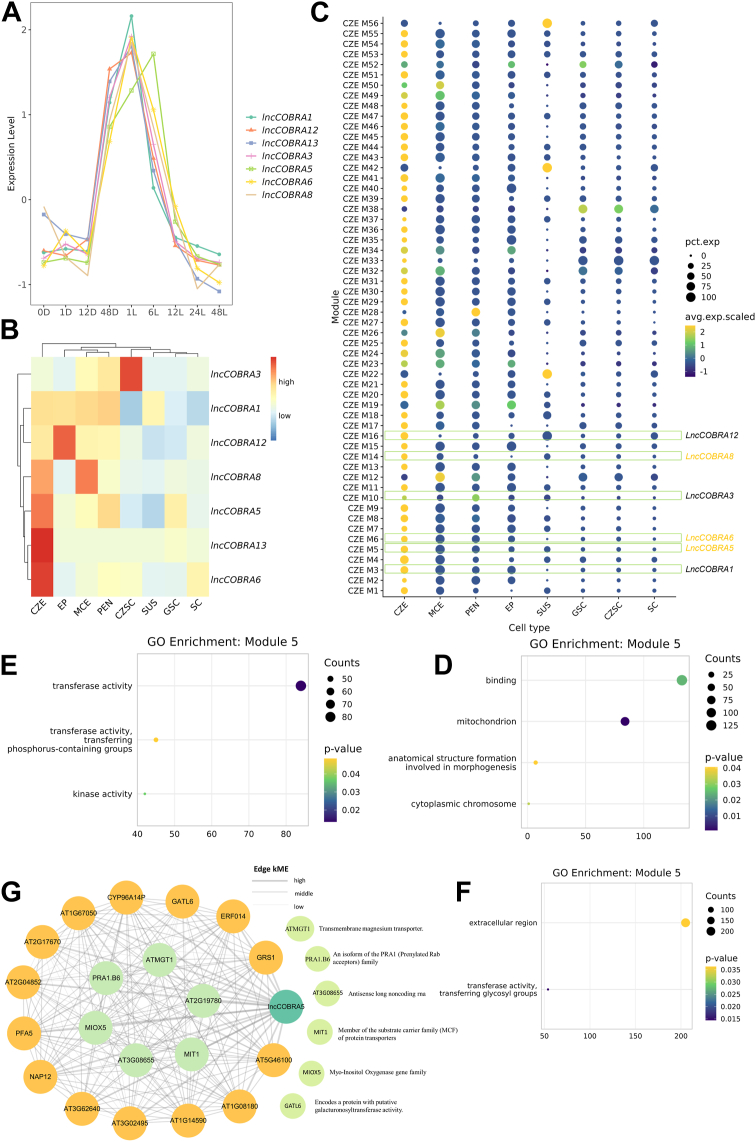
**Prediction of *lncCOBRAs* function through single-cell co-expression network.***A*, expression pattern of *lncCOBRAs* in bulk RNA-seq during the seed germination process. *B*, expression heatmap of *lncCOBRAs* in scRNA-seq across different cell types. *C*, gene co-expression network of the chalazal endosperm (CZE). Bubble size represents the proportion of expression of module eigengenes (MEs) across different cell types, while bubble color indicates the average expression level of MEs in each cell type. The module marked with a *green box* contains *lncCOBRAs*, and the *yellow-highlighted lncCOBRAs* are specifically expressed in CZE. *E*–*F*, enrichment analysis results for modules containing *lncCOBRA5*, *lncCOBRA6*, and *lncCOBRA8*, respectively. *G*, co-expression network of the module containing *lncCOBRA5*, composed of the top 20 genes most strongly correlated with *lncCOBRA5.* The central node represents the five genes with the strongest co-expression relationships.

Single-cell RNA sequencing enables the investigation of lncRNAs expression dynamics at the single-cell level, offering a more precise understanding of their functional roles (18). A previous study identified a conserved class of long non-coding RNAs (lncRNAs), termed *lncCOBRAs*, which were initially discovered in *A. thaliana* and exhibit high sequence homology with lncRNAs in *Brassica rapa*. Many of these *lncCOBRAs* harbor snoRNA domains (42). Notably, *lncCOBRA1* has been experimentally validated to play a role in seed development and plant growth (42). However, the functional characterization of other *lncCOBRAs* remains limited. Based on published RNA-seq data (42), we found that these *lncCOBRAs* display highly similar expression dynamics during seed germination. Specifically, when seeds transition from dark to light conditions, all *lncCOBRAs* exhibit a marked increase in expression, peaking at 1 hour after light exposure (Fig. 6*A*), suggesting a potential role in light-responsive germination.

To further explore their biological relevance, we analyzed single-cell RNA-seq data from developing seeds (43). The results revealed strong cell-type-specific expression patterns among *lncCOBRAs*. For example, *lncCOBRA5*, *lncCOBRA6*, *lncCOBRA8*, and *lncCOBRA13* were exclusively expressed in the chalazal endosperm (Fig. 6*B*), suggesting potential regulatory roles in these specialized cells. In contrast, *lncCOBRA1* showed relatively broad expression across seed tissues, with the exception of the seed coat, and lacked clear cell-type specificity (Fig. 6*B*). Overall, most *lncCOBRAs* exhibited specific expression patterns, highlighting their likely functional specialization during seed development.

Co-expression networks are powerful tools for identifying functionally related gene modules, which often correspond to specific biological processes (27). Therefore, based on gene expression profiles from chalazal endosperm cells that specifically express *lncCOBRAs*, we constructed a gene co-expression network and identified 56 distinct modules. Most of the genes within these modules exhibited their highest expression levels in the chalazal endosperm (Fig. 6*C*). Interestingly, six out of the seven *lncCOBRAs* were assigned to different co-expression modules, with the exception of *lncCOBRA13*, suggesting that these lncRNAs may participate in diverse functional pathways (Fig. 6*C*). We focused on *lncCOBRA5*, *lncCOBRA8*, and *lncCOBRA6* due to their specific expression in chalazal endosperm cells and performed functional enrichment analysis on their corresponding co-expression modules. The analysis revealed that the module containing *lncCOBRA8* (module 14) was significantly enriched in glycosyltransferase activity, while the module containing *lncCOBRA5* (module 5) was enriched in phosphorus-containing group transferase activity (Fig. 6, *E* and *F*). Moreover *lncCOBRA6* (module 6) was associated with morphogenesis-related functions. These findings suggest that the *lncCOBRAs* may play distinct and specialized roles in the biological processes of chalazal endosperm cells.

To further investigate the potential functions of these *lncCOBRAs*, we extracted sub-networks centered on the three aforementioned *lncCOBRAs*. The results showed that *lncCOBRA5* exhibited stronger correlations with its neighboring genes than *lncCOBRA8* and *lncCOBRA6*. Therefore, we focused our subsequent analysis on the network surrounding *lncCOBRA5*. Notably, the top three genes most highly correlated with *lncCOBRA5* were *ATMGT1*, *PRA1.B6*, and AT2G19780, all of which are closely associated with transmembrane transport. *ATMGT1* encodes a magnesium ion transporter that shows high homology across plant species (44); *PRA1.B6* is a member of the PRA protein family and is involved in the regulation of vesicle trafficking regulation (45); and AT2G19780 has been shown to contain multiple structural domains, including transmembrane regions (46). In addition, since module 5—the module containing *lncCOBRA5*—was significantly enriched in phosphorus-containing group transferase activity, a function often associated with ATP consumption and phosphorylation events during membrane transport (47), we hypothesize that *lncCOBRA5* may coordinate with protein-coding genes to regulate transmembrane transport processes. Although further experimental evidence is required to validate this hypothesis, our findings provide new insights and methodological avenues for studying *lncCOBRAs*. Moreover, this case highlights the importance and utility of the extensive co-expression networks constructed in our database.

## Discussion

An increasing number of studies have demonstrated that lncRNAs play crucial regulatory roles in various biological processes (5). With the continuous advancement of transcriptomic technologies, researchers are now able to explore the expression and functions of lncRNAs at the single-cell level (18). Single-cell transcriptomic technologies preserve cellular heterogeneity, offering unique advantages for studying lncRNAs, and have already led to substantial achievements in the animal field (19, 21). To promote in-depth research on plant lncRNAs, we have developed a comprehensive database platform based on plant single-cell transcriptome data. This platform focuses on the expression and regulatory characteristics of lncRNAs, enabling users to systematically investigate lncRNAs at single-cell resolution and providing a novel data resource for the field.

The database provides a comprehensive collection of lncRNA expression profiles. Users can explore the expression patterns of specific lncRNAs across different tissues and cell types *via* a search function. Compared to recently developed plant single-cell transcriptome databases PscLncRNAs (26), our platform not only integrates datasets from the same tissues to comprehensively investigate lncRNA expression and regulation across various tissues and cell types, but also performs independent analyses for each sample. This approach accounts for potential differences among samples, such as developmental stage and environmental conditions, and better reveals the expression characteristics of lncRNAs under diverse biological contexts. The expression features of lncRNAs are visualized using both violin plots and UMAP plots. Violin plots clearly show the distribution of lncRNAs expression across different cell types, while UMAP plots intuitively present the spatial expression patterns of lncRNAs, even for those expressed in only a few cells. In addition, users can compare lncRNA expression across different tissues or among samples from the same tissue, helping to uncover their potential functions under specific conditions.

The database also includes a set of newly predicted marker genes, comprising 2177 lncRNAs with cell type-specific expression (Figs. 2*B* and 4, *A* and *B*). Marker genes are essential tools for cell type annotation, and recent studies have shown that lncRNAs alone can be used to accurately identify cell types in animals (48), further supporting their potential as marker genes. Our analysis reveals that a subset of these predicted lncRNAs exhibits expression patterns similar to those of coding genes, such as being specifically expressed in xylem cells (Fig. 4*C*). This suggests that they have potential in cell type identification and may be comparable to protein-coding genes. The database provides visualizations of these lncRNA markers, facilitating further functional investigation. Notably, while this platform primarily focuses on lncRNAs, it also provides protein-coding genes to support more comprehensive systems biology analyses.

To further uncover the potential functions of lncRNAs, we constructed cell type-specific co-expression networks of lncRNAs and protein-coding genes based on single-cell transcriptomic data. Compared to traditional bulk RNA-seq data, single-cell data avoid the averaging effect of expression values, making them particularly suitable for studying low-abundance or specifically expressed lncRNAs (18). This advantage enabled us to construct more comprehensive co-expression networks. The database includes co-expression networks for over 200 cell types, encompassing thousands of functional modules and integrating approximately 13,591 lncRNAs (Fig. 2*C*). Interestingly, many of these lncRNAs act as hub genes within the networks (Fig. 2*D*), suggesting that they may play central roles in regulating module functions. Additionally, through the co-expression network, we predicted the functions of *lncCOBRAs*. Notably, the module containing *lncCOBRA5* is enriched in genes related to transmembrane transport (Fig. 6*G*) and is significantly associated with phosphotransferase activity in the functional enrichment analysis (Fig. 6*E*). Given that transmembrane transport is an energy-intensive process closely tied to ATP metabolism, and phosphorylation is a key mechanism for ATP energy transfer (49), we speculate that *lncCOBRA5* may coordinately regulate transmembrane transport. Although this hypothesis requires further experimental validation, this case highlights the application potential of co-expression networks in lncRNAs functional prediction and further emphasizes the value of our database in plant lncRNAs research.

It should be noted that the lncRNAs currently included in the database are derived from existing next-generation sequencing data and do not yet incorporate newly identified transcripts. Due to its preservation of cellular heterogeneity, single-cell transcriptomics holds promise for identifying novel lncRNAs that are difficult to detect using bulk RNA-seq (18). For example, in mammals, a study identified 30.4% novel lincRNAs in 359 cardiomyocyte nuclei that could only be detected *via* scRNA-seq (50). Our analysis shows that some lncRNAs undetectable in root RNA-seq data exhibit expression in single-cell transcriptomes (Fig. 5*C*) and display specific expression in particular cell types (Fig. 5, *B* and *C*), further supporting the feasibility of using single-cell data for the discovery of novel lncRNAs, especially for locally expressed lncRNAs. However, most current single-cell platforms applied to plants, such as 10× Genomics, are based on non-full-length library preparation strategies and are not suitable for transcript assembly. With the advancement of full-length single-cell transcriptomics, more novel plant lncRNAs may be identified at single-cell resolution in the future, deepening our understanding of their regulatory mechanisms.

To support ongoing advances in plant single-cell research, we will continue to update and improve our database. Future developments will include the integration of data from additional plant species, the identification of novel lncRNAs using single-cell technologies, and the enhancement of database functionality—such as the development of intuitive visualization and analysis tools to facilitate deeper exploration of lncRNAs. Overall, this platform offers a valuable resource and analytical toolkit for plant lncRNA research, enabling systematic investigation of their expression and regulatory roles at single-cell resolution and contributing to the advancement of plant functional genomics.

## Experimental procedures

### ScRNA-seq and bulk RNA-seq data collection

We conducted a search on PubMed and Google using terms such as “single cell,” “scRNA-seq,” and “single cell transcriptomics” to identify relevant published studies. We filtered for plant-related articles and excluded those lacking publicly available data. After screening, we retained studies that employed the 10× Genomics or Smart-seq platforms, resulting in a total of 39 studies covering eight plant species (Table S1). Data collection was completed by December 2022. In addition, bulk RNA-seq data were compiled from previous studies, including Arabidopsis root tips at 7 days as well as other tissues such as stem, leaf, and shoot apex. Details of these datasets are provided in Table S3.

### LncRNAs references construction

While we have collected many single-cell transcriptomics datasets, many of them are not suitable for novel lncRNA prediction due to the lack of full-length transcript coverage. Therefore, we supplemented our collection with lncRNAs predicted from bulk RNA-seq data available in databases such as PLncDB, GreeNC, and species-specific resources like TAIR (https://www.arabidopsis.org/) and RGAP (https://rice.uga.edu/). Notably, PLncDB integrates lncRNAs from diverse sources.

After obtaining candidate lncRNAs, we prioritized entries from TAIR and RGAP. For lncRNAs from other sources, we performed sequence alignment against the reference genome to determine their genomic context—whether they are located in intergenic regions, intronic regions, or on the antisense strand of coding genes. We used the proportion of intergenic lncRNAs as a proxy for source credibility, with higher proportions suggesting greater reliability. Based on this evaluation, we ranked the sources and removed redundant lncRNAs, giving preference to those from the most reliable datasets.

After removing redundant entries, we performed another positional comparison between the lncRNAs and the reference genome. Considering that lncRNAs completely overlapping with protein-coding genes may lead to inaccurate quantification, we mainly retained those located in intergenic regions and antisense lncRNAs. This is because the 10× Genomics single-cell transcriptome libraries are strand-specific, which ensures that the quantification of antisense lncRNAs is not affected by mRNA expression levels. Statistics on the collected lncRNAs are provided in Table S4.

### RNA-seq data processing

After quality control, reads were aligned to the reference genome TAIR 10 (which included additional lncRNAs) using HISAT2 (v2.2.1) (51). Quantification was then performed with StringTie (v2.1.7) (52). Data normalization across different samples was achieved using “data_norm” function in GCEN (v 0.6.3) (53).

### ScRNA-seq data processing

The raw scRNA-seq data in FASTQ format were aligned to a reference genome containing numerous lncRNAs using the CellRanger pipeline (v7.0.1, 10× Genomics), with default parameters for barcode assignment and unique molecular identifier (UMI) counting.

Filtered count matrices, generated by the CellRanger pipeline using identical criteria for both mRNAs and lncRNAs, were further analyzed with the Seurat package (v4.3.0) (54). Cells with fewer than 200 detected genes and genes expressed in fewer than three cells were excluded from downstream analyses. Data normalization was performed using the “LogNormalize” method in the NormalizeData function. Variably expressed genes were identified using the “vst” method in the FindVariableFeatures function, and the top 2000 variable genes were selected for downstream analysis. Data scaling was then conducted using the ScaleData function.

For certain tissues, multiple datasets were collected, and we performed both individual analyses and integrative analyses of these datasets from the same tissue. Batch effects were removed using the Harmony algorithm (55). Principal component analysis (PCA) was performed using the ‘‘RunPCA’’ function, computing the top 50 principal components based on the top 2000 variable genes. Clustering was then conducted using Seurat’s “FindNeighbors” and “FindClusters” functions. The “FindNeighbors” function calculated the k-nearest neighbor graph to capture cell relationships, and “FindClusters” was used to identify distinct cell clusters based on shared nearest neighbors (SNN), with a resolution of approximately 0.8 (this parameter may vary across datasets).

To visualize the clustering results, both UMAP (Uniform Manifold Approximation and projection) and t-SNE (t-Distributed Stochastic Neighbor Embedding) were employed. For cell type annotation, known marker genes were used to assess gene expression patterns across clusters. Additionally, AUCell (v1.16.0) (56) was used for gene set scoring, aiding in the accurate identification of cell types across the clusters.

### Marker gene identification

The curated marker genes were primarily gathered from recent single-cell studies and manually selected based on experimental validation or strong computational evidence, with an emphasis on well-characterized markers. To further enrich the dataset, we also integrated experimentally supported entries from the “Experiment” section of the PMCBD database, while rigorously removing duplicates across sources to ensure a high-quality, non-redundant marker gene set.

For the identification of novel marker genes, markers for each cell cluster were determined using the “FindAllMarkers” function (with min.pct = 0.1 and logfc.threshold = 0.25). A *p*-value adjustment threshold of 0.005 was applied to retain high-confidence markers. We further classified these markers based on their expression specificity: those with pct.1 > 0.25 and a pct.1/pct.2 ratio greater than 2.5 were defined as Degree 1 (D1) markers, representing high-confidence cluster-specific markers, while others were labeled Degree 2 (D2) markers. All novel and well-characterized markers are accessible through the website we developed.

### Cell–cell communication inferring

Based on the processed scRNA-seq data, ligand-receptor pairs were extracted from PlantPhoneDB (29). The cell–cell ligand–receptor interactions were scored using the "LRscore" function from PlantPhoneDB (veision 1.0.0). Finally, the results were visualized using a chord diagram.

### Cell type-specific network analysis

Based on cell type annotation, we constructed cell type-specific co-expression networks between lncRNAs and protein-coding genes using hdWGCNA (v0.2.26) for each cell population. A minimum of 100 cells was required to construct each co-expression network. The soft threshold was selected as the lowest value that yielded a Scale-Free Topology Model Fit of at least 0.8. If no soft threshold reached this value, the one corresponding to the maximum Scale-Free Topology Model Fit was chosen. To present the results of each module more intuitively and effectively, we used bubble plots to visualize the overall gene expression patterns within each module across cell groups. In these plots, bubble size indicates the relative proportion of module eigengene (ME) expression across different cell types, while bubble color represents the average ME expression level in each cell type. This visualization approach facilitates an initial assessment of modules that may play important roles in specific cell types.

### GO enrichment analysis

By performing functional enrichment analysis for each cell cluster, we can gain deeper insights into their biological functions and subsequently determine the cell types. Based on the scRNA-seq clustering results, the differentially expressed genes for each cluster are input into the functional enrichment analysis, which is carried out using the clusterProfiler package (57).

## Data availability

All datasets used in this study were obtained from publicly available databases. Detailed information on data sources is provided in Tables S1 and S3.

## Supporting information

This article contains supporting information.

## Conflict of interest

The authors declare that they have no conflicts of interest with the contents of this article.
