# Supplementary material for: GreenCells: A comprehensive resource for single-cell analysis of plant lncRNAs
Source: J Biol Chem. 2025 Sep 3;301(10):110678. doi: 10.1016/j.jbc.2025.110678 (PMC12509759; doi:10.1016/j.jbc.2025.110678)
Supplement: Supplemental Information [file mmc1.docx]

**GreenCells: A comprehensive resource for single-cell analysis of plant lncRNAs**

Changxiong Wu^1,2,6^, Jiazhi Liu^4,5,6^, Yan Li^2,5,6^, Wenjing Yang^2^, Jie Wang^3,*^, Changning Liu1,^2,*^

1 School of Life Sciences, Division of Life Sciences and Medicine, University of Science and Technology of China, Hefei 230027, China

2 CAS Key Laboratory of Tropical Plant Resources and Sustainable Use, Yunnan Key Laboratory of Crop Wild Relatives Omics, Xishuangbanna Tropical Botanical Garden, Chinese Academy of Sciences, Kunming 650223, China

3 Department of Chromosome Biology, Max Planck Institute for Plant Breeding Research, Carl-von-Linne-Weg 10, Cologne 50829, Germany

^4^ Germplasm Bank of Wild Species & Yunnan Key Laboratory of Crop Wild Relatives Omics, Kunming Institute of Botany, Chinese Academy of Sciences, Kunming, Yunnan 650201, China

^5^ University of Chinese Academy of Sciences, Beijing 100049, China

^6^ These authors contributed equally to this article.

^*^**Correspondence: Jie Wang (jiewang@mpipz.mpg.de), Changning Liu (liuchangning@xtbg.ac.cn)**

## S**upporting information**

**Figure S1.** LncRNAs characters.

**Figure S2.** Extended statistics of the database.

**Figure S3.** Expression pattern of hub genes.

**Figure S4.** Clustering and annotation map of integrated root data.

**Figure S5.** Marker correlation between lncRNAs and coding genes.

**Figure S6.** Heatmap of scRNA-seq-specific genes in root tip.

**Figure S7.** Heatmap of root tip-specific genes in scRNA-seq.

**Table S1 (Excle).** ScRNA-seq data source.

**Table S2 (Excle).** Marker genes used for correlation analysis.

**Table S3.** Reference information for RNA-seq data.

**Table S4.** LncRNA database reference.

**
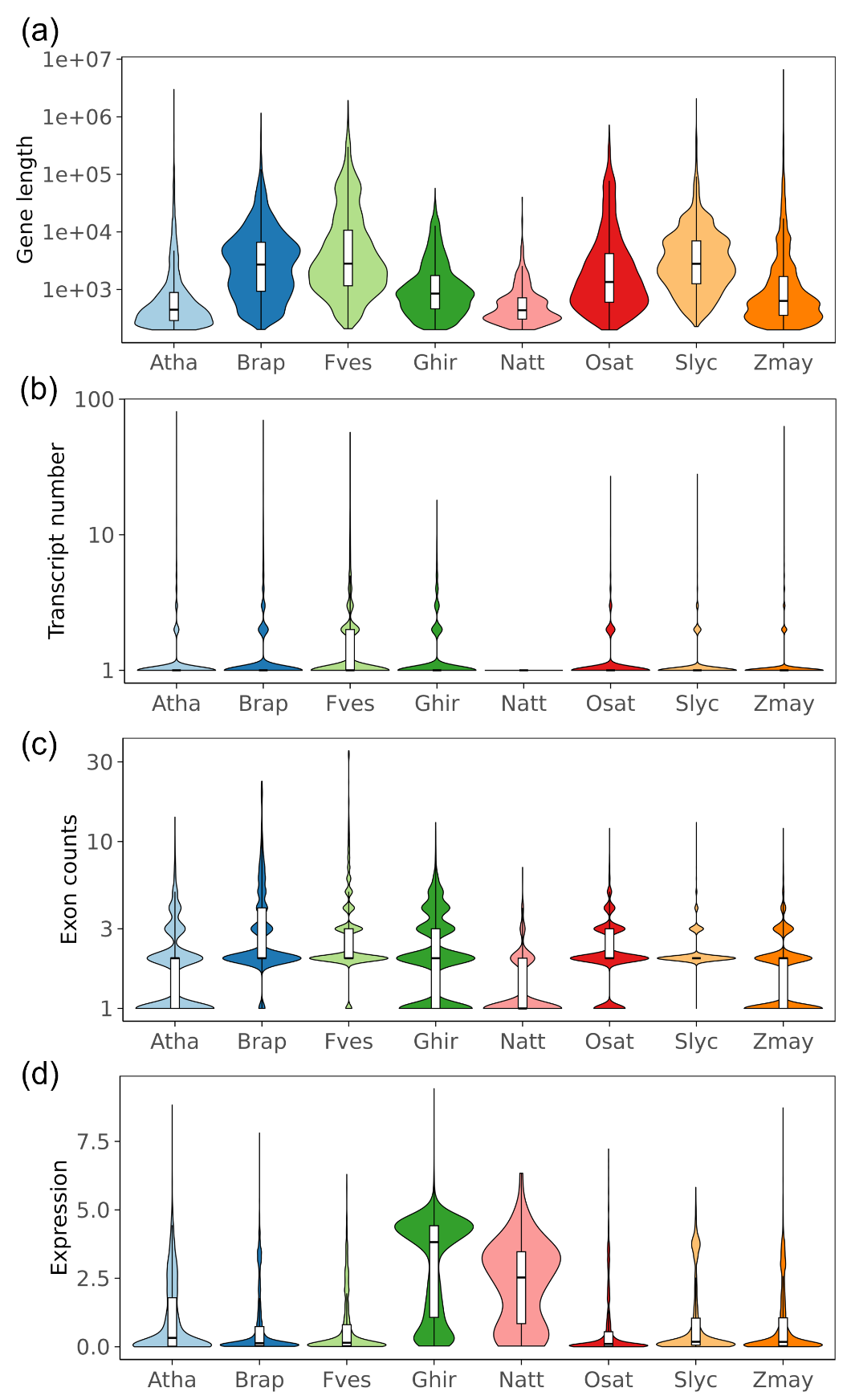
**

**Figure S1. LncRNAs characters.**

(a) Length distribution of lncRNA genes in each species.

(b) Transcript count distribution per lncRNA gene in each species.

(c) Exon count distribution per lncRNA transcript in each species.

(d) LncRNA expression profiles across species were computed as the mean expression within the top 1% of cells with the highest expression levels in each species. Expression values were normalized using Seurat’s “LogNormalize” method, which performs library size normalization followed by log-transformation using log1p.


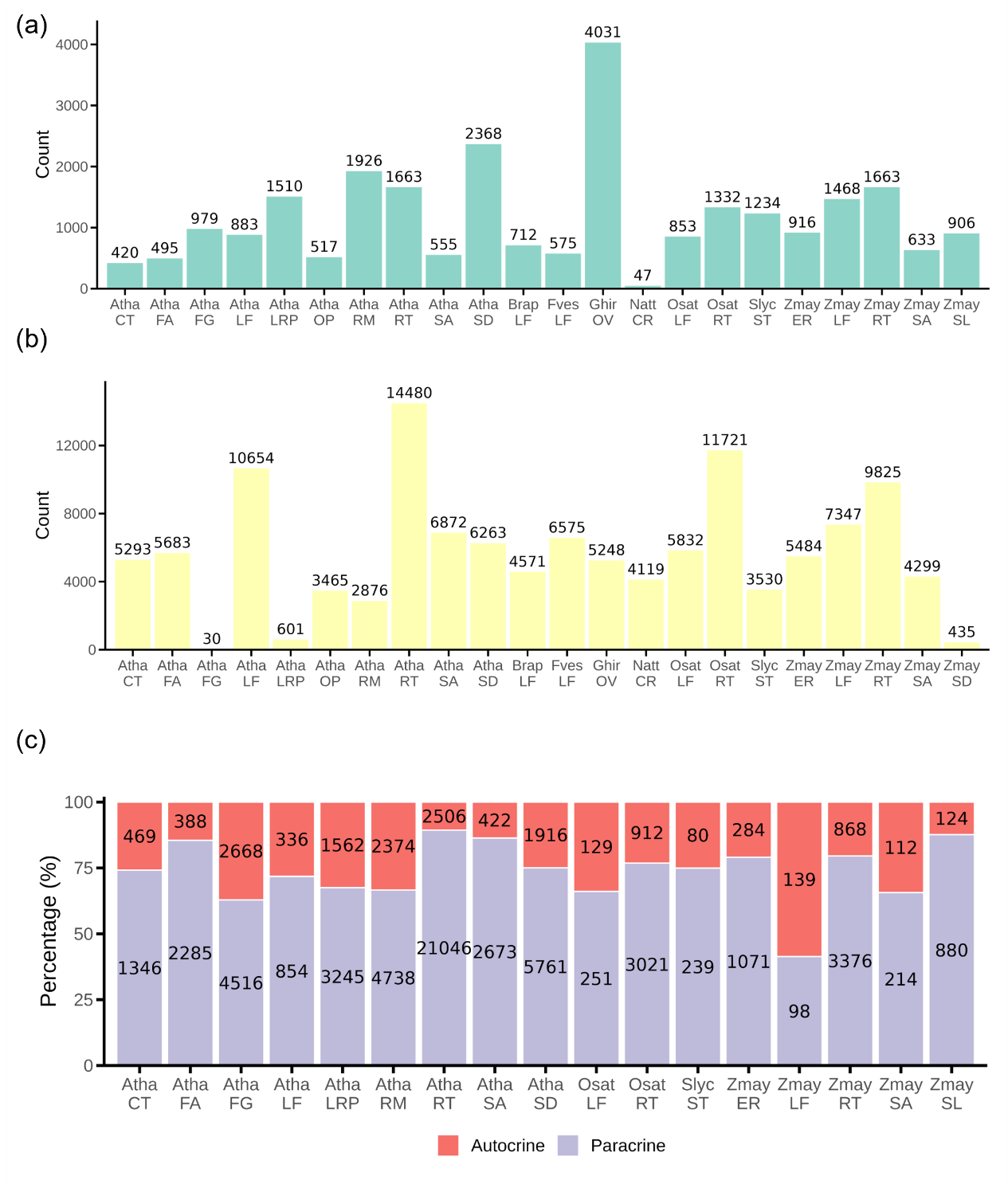


**Figure S2. Extended statistics of the database.**

(a) Expression counts of protein-coding genes across different tissues.

(b) Counts of predicted protein-coding marker genes.

(c) Statistics on the number of ligand-receptor pairs involved in cell–cell communication, including both paracrine and autocrine interactions.

**
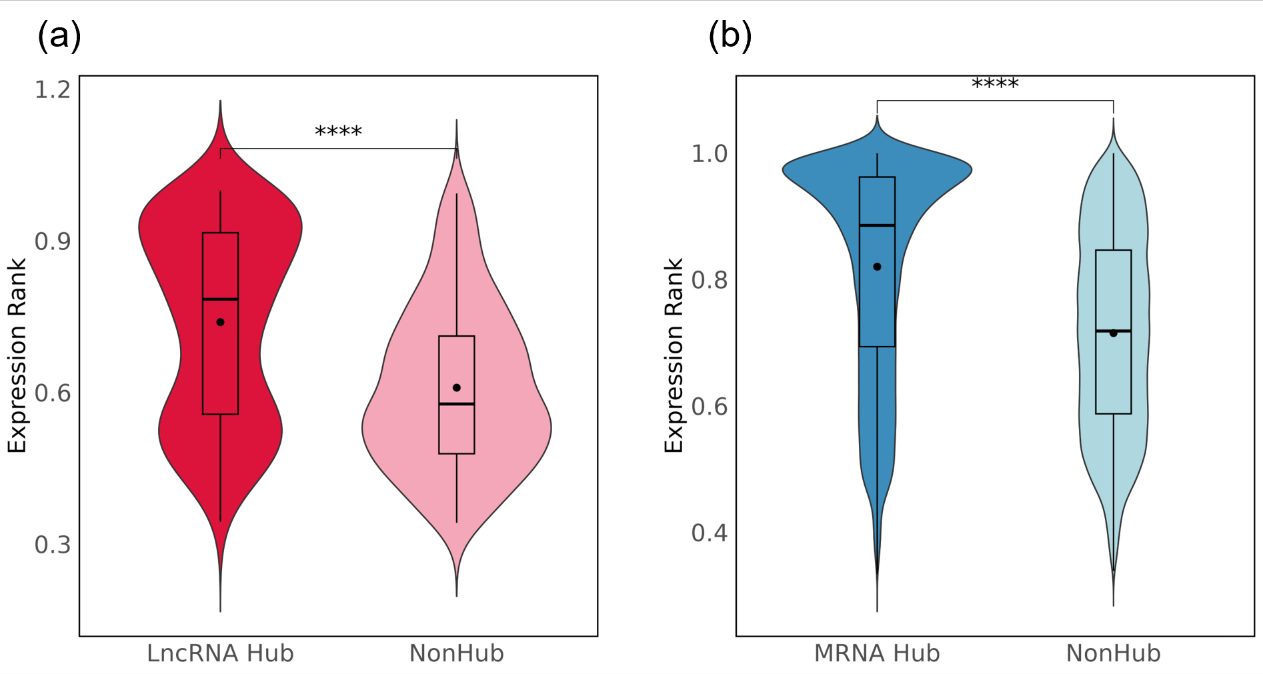
**

**Figure S3. Expression pattern of hub genes.**

The expression rank of hub genes and randomly selected non-hub genes, where higher values indicate higher expression levels. Panel (a) shows lncRNA hubs compared to non-hub lncRNAs, and panel (b) shows coding hub genes compared to non-hub coding genes. Statistical significance was assessed using the Wilcoxon rank-sum test (****, p < 0.0001). The dots represent the overall mean expression.


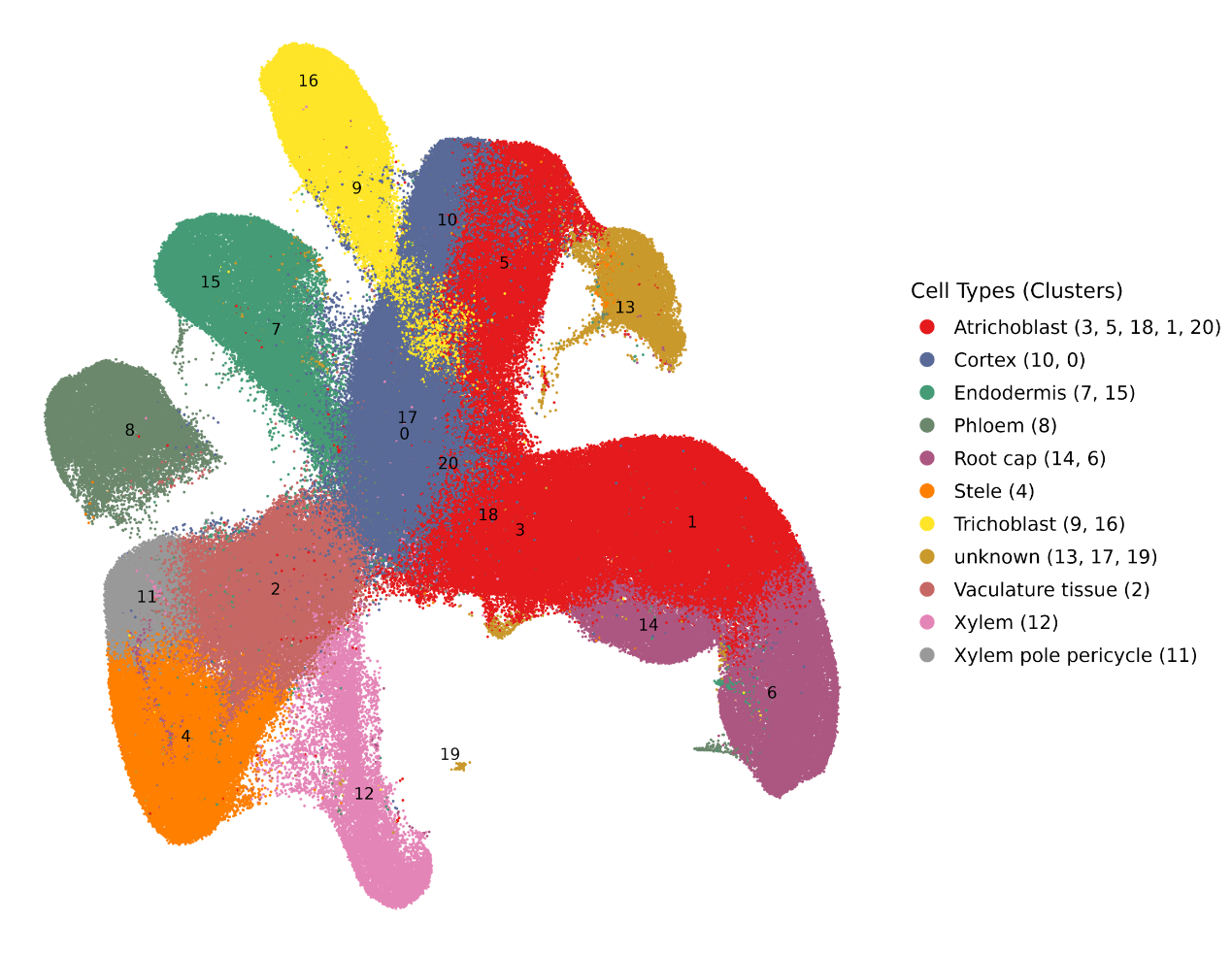


**Figure S4. Clustering and annotation map of integrated root data.**

Cluster and annotation map of the integrated Arabidopsis root dataset, related to the figure 4, with data sources provided in Table S1.


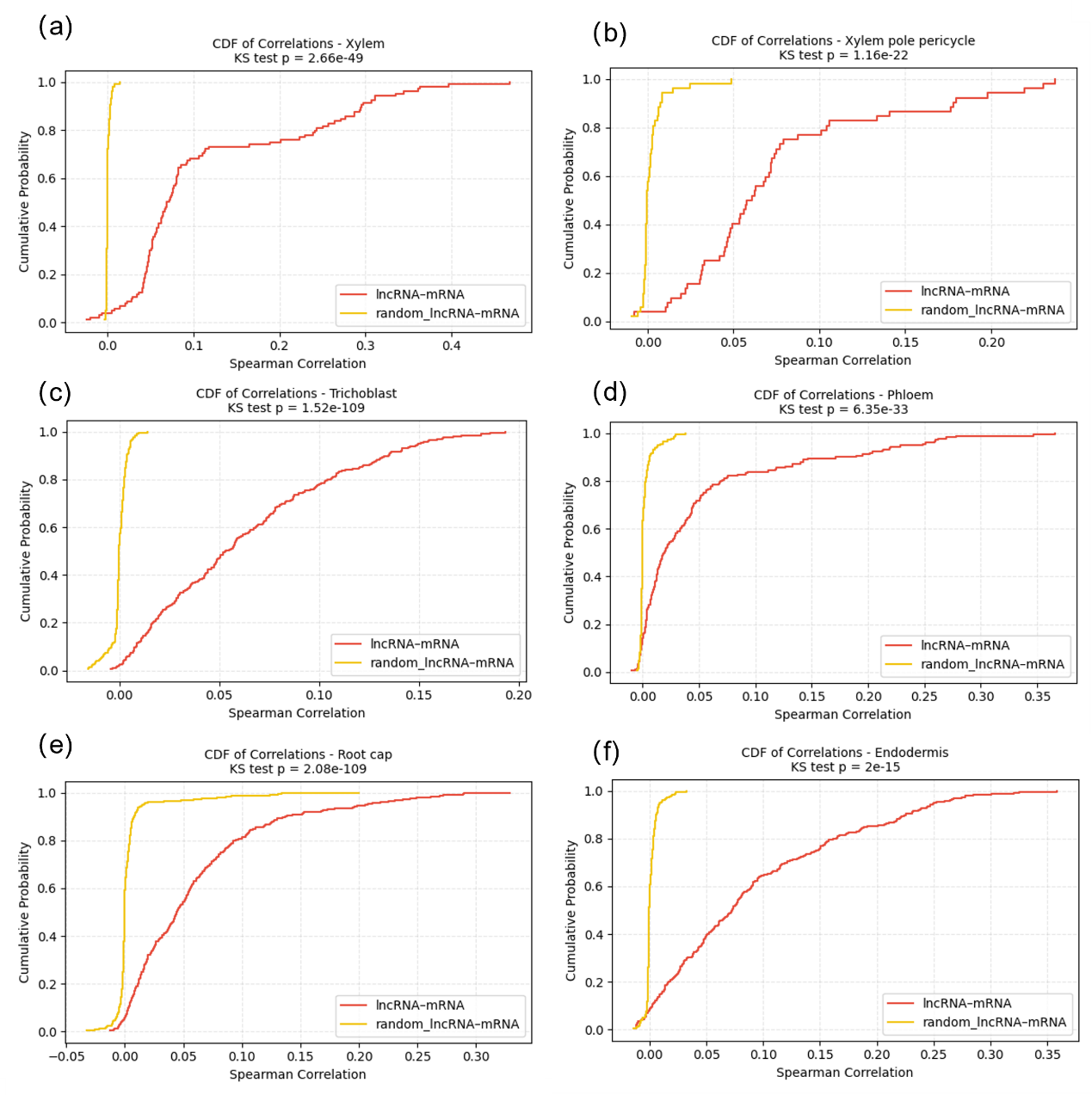


**Figure S5. Marker correlation between lncRNAs and coding genes.**

Correlation of expression between predicted lncRNA markers and known protein-coding marker genes, calculated across all single cells. Red lines indicate predicted lncRNA markers, while yellow lines represent randomly selected non-marker lncRNAs. Panels (a–e) display results for the following cell types: Xylem, Xylem pole pericycle, Trichoblast, Phloem, Root cap, and Endodermis.


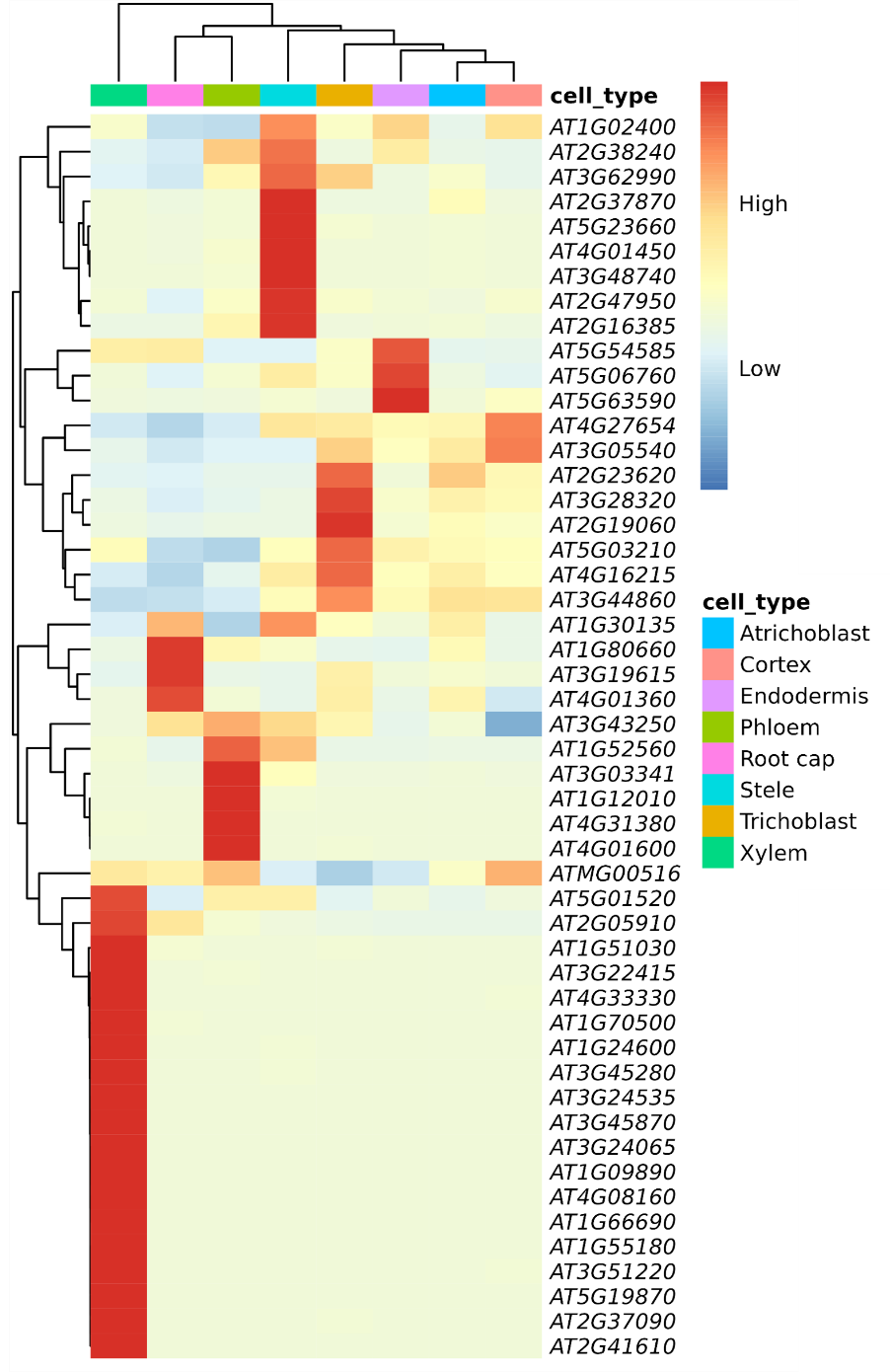


**Figure S6.** **Heatmap of scRNA-seq-specific genes in root tip.**

Heatmap showing the expression patterns of protein-coding genes that are absent in bulk root tip data of *Arabidopsis thaliana* but present in scRNA-seq data. The genes in the figure are the top 50 based on average expression level after been normalized.


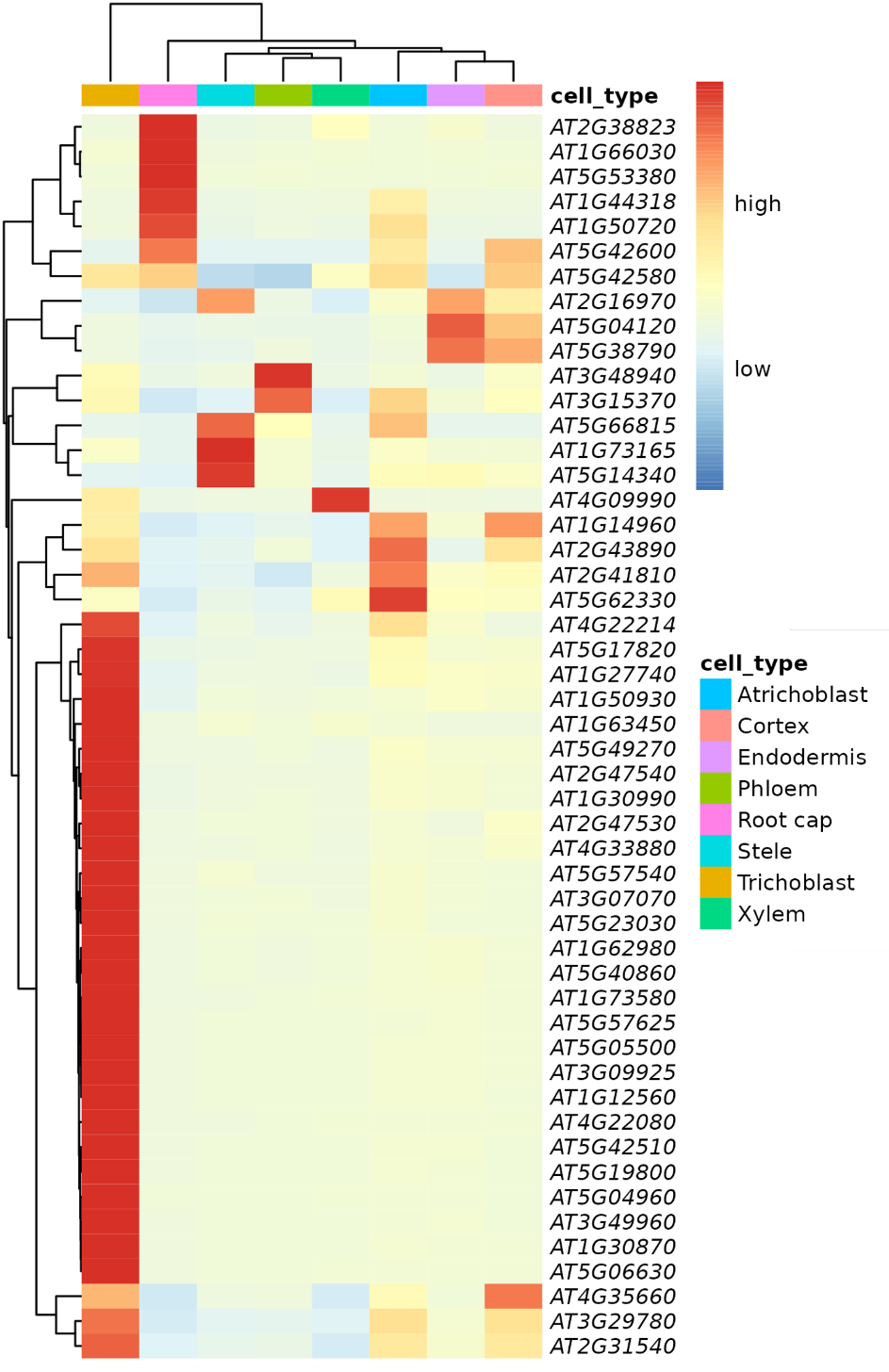


**Figure S7. Heatmap of root tip-specific genes in scRNA-seq.**

Heatmap of expression patterns of bulk root tip-specific protein-coding genes across cell types in scRNA-seq data of *Arabidopsis thaliana*. The genes in the figure are the top 50 based on average expression level after been normalized.

**Table S3. Reference information for RNA-seq data.**

| Species | Accession | Ecotype | Tissue | PMID |
| --- | --- | --- | --- | --- |
| *Arabidopsis thaliana* | PRJNA478919 | Col-0 | root tip | 30061750 |
| *Arabidopsis thaliana* | PRJNA478919 | Col-0 | root tip | 30061750 |
| *Arabidopsis thaliana* | PRJNA478919 | Col-0 | root tip | 30061750 |
| *Arabidopsis thaliana* | CRA003591 | Col-0 | flower | 35590257 |
| *Arabidopsis thaliana* | CRA003591 | Col-0 | silique | 35590257 |
| *Arabidopsis thaliana* | CRA003591 | Col-0 | stem | 35590257 |
| *Arabidopsis thaliana* | CRA003591 | Col-0 | leaf | 35590257 |
| *Arabidopsis thaliana* | PRJNA261433 | Col-0 | shoot | 26945048 |
| *Arabidopsis thaliana* | PRJNA261433 | Col-0 | shoot | 26945048 |
| *Arabidopsis thaliana* | PRJNA261433 | Col-0 | shoot | 26945048 |

**Table S4. LncRNA database reference.**

| Species | Source | Genome | Transcripts nums | Genes nums |
| --- | --- | --- | --- | --- |
| *Arabidopsis thaliana* | PLncDB/  CANTATAdb/TAIR | TAIR10 | 6,462 | 4,968 |
| *Brassica rapa* | PLncDB | GCF_000309985.2 | 12,101 | 7,983 |
| *Fragaria vesca* | PLncDB/NCBI | GCF_000184155.1 | 10,720 | 6,253 |
| *Gossypium hirsutum* | https://doi.org/10.3389/fgene.2018.00690 | GCF_000987745.1 | 10,315 | 7,156 |
| *Nicotiana attenuata* | GreeNC | NIATTr2 | 1,314 | 1,314 |
| *Oryza sativa* | PLncDB/RGAP | MSU_osa1r7 | 4,340 | 4,340 |
| *Solanum lycopersicum* | PLncDB/ITAG | ITAG4.0 | 7,826 | 6,684 |
| *Zea mays* | PLncDB | B73_RefGen_v4 | 24,440 | 21,034 |
